# Prognostic Nomograms for Predicting Survival and Distant Metastases in Locally Advanced Rectal Cancers

**DOI:** 10.1371/journal.pone.0106344

**Published:** 2014-08-29

**Authors:** Junjie Peng, Ying Ding, Shanshan Tu, Debing Shi, Liang Sun, Xinxiang Li, Hongbin Wu, Sanjun Cai

**Affiliations:** 1 Department of Colorectal Surgery, Fudan University Shanghai Cancer Center, Shanghai, China; 2 Department of Oncology, Shanghai Medical College, Fudan University, Shanghai, China; 3 Department of Biostatistics, University of Pittsburgh, Pittsburgh, Pennsylvania, United States of America; 4 Department of Statistics, University of Pittsburgh, Pittsburgh, Pennsylvania, United States of America; 5 School of Science and Technology, Georgia Gwinnett College, Atlanta, Georgia, United States of America; The University of Texas MD Anderson Cancer Center, United States of America

## Abstract

**Aim:**

To develop prognostic nomograms for predicting outcomes in patients with locally advanced rectal cancers who do not receive preoperative treatment.

**Materials and Methods:**

A total of 883 patients with stage II–III rectal cancers were retrospectively collected from a single institution. Survival analyses were performed to assess each variable for overall survival (OS), local recurrence (LR) and distant metastases (DM). Cox models were performed to develop a predictive model for each endpoint. The performance of model prediction was validated by cross validation and on an independent group of patients.

**Results:**

The 5-year LR, DM and OS rates were 22.3%, 32.7% and 63.8%, respectively. Two prognostic nomograms were successfully developed to predict 5-year OS and DM-free survival rates, with c-index of 0.70 (95% CI = [0.66, 0.73]) and 0.68 (95% CI = [0.64, 0.72]) on the original dataset, and 0.76 (95% CI = [0.67, 0.86]) and 0.73 (95% CI = [0.63, 0.83]) on the validation dataset, respectively. Factors in our models included age, gender, carcinoembryonic antigen value, tumor location, T stage, N stage, metastatic lymph nodes ratio, adjuvant chemotherapy and chemoradiotherapy. Predicted by our nomogram, substantial variability in terms of 5-year OS and DM-free survival was observed within each TNM stage category.

**Conclusions:**

The prognostic nomograms integrated demographic and clinicopathological factors to account for tumor and patient heterogeneity, and thereby provided a more individualized outcome prognostication. Our individualized prediction nomograms could help patients with preoperatively under-staged rectal cancer about their postoperative treatment strategies and follow-up protocols.

## Background

Colorectal cancer is the most commonly diagnosed gastrointestinal malignancy in the world. As most of patients with rectal cancer present with locally advanced disease at diagnosis, neoajuvant chemoradiation is the standard recommendation to improve patients’ outcomes including quality of life. Compared to colon cancer, treatment is more heterogeneous in rectal cancer. In real clinical practice, approximately 20–50% of patients with stage II–III rectal cancer in North America receive definitive surgery prior to adjuvant treatment [1], [2], and the proportion is even higher in Asia [3]. The reasons for not giving neoadjuvant therapy may be multifarious. Although neoadjuvant chemoradiotherapy (CRT) has been confirmed to improve local control for locally advanced rectal cancer, its efficacy in preventing distant metastases and improving OS remains controversial [4]. Because preoperative CRT is associated with increased complications compared to surgery alone, we sought to characterize patients with locally advanced rectal cancer who were adequately treated with surgery followed by adjuvant chemotherapy[5]–[7].

Currently, the TNM stage system from the American Joint Commission on Cancer (AJCC) and the International Union Against Cancer [8], [9] is the most reliable prognostic system for all stages of rectal cancer patients with or without preoperative treatment [10], [11]. However, TNM staging does not integrate demographic features like age, or other pathological features like histopathology, perineural invasion, or tumor location, into a patient’s outcome prediction. More individualized outcome prediction models could help physicians advise patients about personalized treatment strategies and follow-up protocols.

Developing a nomogram for prognosis or treatment prediction has been considered helpful in individualized medicine and successful applications have been utilized in many malignancies[12]–[15]. This statistically based tool provides a predicted probability of a specific outcome, using a combined set of proven or potential prognostic factors. Recently, a nomogram was developed to predict outcomes of locally advanced rectal cancers with preoperative radiotherapy or CRT [16]. However, due to changes in pathological features after preoperative treatment, this nomogram only applies to patients who receive preoperative treatment. Our study was designed to develop prognostic nomograms for patients with locally advanced rectal cancer who did not receive preoperative treatment.

## Materials and Methods

### Ethics

A retrospective study was conducted at the Fudan University Shanghai Cancer Center. This study was approved by the Fudan University Shanghai Cancer Center Institutional Ethics Committee. According to hospital routine, patients are asked to provide a written informed consent after their admission that their clinical and outcome information will be used in future scientific studies. Patients’ records and follow-up information were anonymized and de-identified prior to analysis. The institutional Ethics Committee approved the exception of informed consent if informed consent could not be obtained due to patients’ death or lost of follow-up in our institutional database.

### Patient Population

All patients with AJCC stage II–III (restaged according to 7^th^ Edition) [8] rectal cancers were collected from the institutional colorectal cancer database. The statistical analyses were performed for patients operated between 1986 and 2005 (N = 833), whose tumors were located within 15 cm from anal verge. Patients who met one of the following criteria were excluded: (1) received preoperative treatment, (2) synchronous distant metastases, (3) surgery without curative intent, and (4) complete loss of follow-up after surgery.

An independent group of patients with stage II–III rectal cancer (N = 84) who were operated between January 2006 and June 2007 were selected for validation (Table 1).

**Table 1:** Characteristics of all patients with locally advanced rectal cancer and outcomes for 833 patients in training group.

	Training Group							Validation Group
Variable	No. (%)	Local Control		Distant Control		Overall Survival		No. (%)
	(n = 833)	5 Year	P-value	5 Year	P-value	5 Year	P-value	(n = 84)
***Demographic Variables***								
Gender								
Male	490 (58.8)	0.770	0.423	0.637	0.006	0.608	0.008	50 (59.5)
Female	343 (41.2)	0.787		0.725		0.682		34 (40.5)
Age, years								
< = 49	262 (31.4)	0.738	0.354	0.649	0.191	0.609	0.097	29 (34.5)
50–69	432 (51.9)	0.802		0.699		0.668		50 (59.5)
> = 70	139 (16.7)	0.772		0.637		0.604		5 (6.0)
***Clinical Variables***								
Tumor location								
Low (<5 cm)	181 (21.7)	0.656	<0.001	0.638	0.221	0.563	0.011	26 (31.0)
Mid (5cm, 10 cm)	570 (68.5)	0.806		0.677		0.651		54 (64.3)
High (>10 cm)	82 (9.8)	0.846		0.719		0.715		4 (4.8)
CEA								
< = 5	406 (48.7)	0.817	0.005	0.696	0.073	0.660	0.062	67 (79.8)
>5	427 (51.3)	0.740		0.650		0.616		17 (20.2)
***Pathological Variables***								
Pathology								
Adenocarcinoma	683 (82.0)	0.773	0.947	0.683	0.184	0.653	0.128	79 (94.0)
MAC or SRC	150 (18.0)	0.792		0.625		0.574		5 (6.0)
Tumor grade								
Low-intermediate grade	708 (85.0)	0.788	0.119	0.681	0.272	0.643	0.356	65 (77.4)
High grade	125 (15.0)	0.713		0.630		0.612		19 (22.6)
pT classification								
T1	13 (1.6)	0.587	0.003	0.539	0.002	0.539	0.001	0 (0)
T2	93 (11.2)	0.83		0.711		0.676		3 (3.6)
T3	351 (42.1)	0.836		0.756		0.725		81 (86.4)
T4	376 (45.1)	0.714		0.589		0.550		0 (0)
pN classification								
N0	324 (38.9)	0.837	<0.001	0.774	<0.001	0.775	<0.001	14 (16.7)
N1a	142 (17.1)	0.753		0.699		0.683		13 (15.5)
N1b	152 (18.2)	0.793		0.638		0.539		24 (28.6)
N2a	112 (13.4)	0.734		0.581		0.548		18 (21.4)
N2b	103 (12.4)	0.619		0.451		0.396		15 (17.8)
Lymphovascular invasion								
Yes	178 (21.4)	0.780	0.956	0.610	0.139	0.547	0.019	42 (50.0)
No	655 (78.6)	0.776		0.691		0.666		42 (50.0)
Perineural invasion								
Yes	111 (13.3)	0.698	0.076	0.544	0.003	0.515	0.002	34 (41.5)
No	722 (86.7)	0.787		0.692		0.656		50 (58.5)
No. of metastatic lymph nodes								
= 0	324 (38.9)	0.775	<0.001	0.837	0.002	0.774	<0.001	14 (16.7)
> = 1	509 (61.1)	0.552		0.735		0.606		70 (83.3)
***Treatment Variables***								
Surgery type								
AR	494 (59.3)	0.832	<0.001	0.689	0.087	0.660	0.012	51 (60.7)
APR	339 (40.7)	0.697		0.649		0.607		33 (39.3)
Adjuvant treatment								
No treatment	252 (30.3)	0.676	<0.001	0.662	0.332	0.633	0.670	16 (19.0)
CT only	277 (33.3)	0.781		0.698		0.663		25 (29.8)
CRT only	123 (14.8)	0.822		0.718		0.612		3 (3.6)
CRT plus CT	181 (21.7)	0.905		0.697		0.628		40 (47.6)

Note: Tumor location was determined the distance from anal verge by preoperative colonoscopy or digital examination.

Abreviations: pT stage, pathological T stage; pN stage, pathological N stage; CEA, carcinoembryonic antigen; MAC, mucinous adenocarcinoma; SRC, signet ring cell carcinoma; AR, anterior resection; APR, abdominoperineal resection; CT, chemotherapy; CRT, chemoradiotherapy.

### Follow-up

According to institutional follow-up protocol, all patients were asked to follow-up every 3–6 months after surgery in the first 3 years, and 6–12 months thereafter in the next two years. Follow-up information was recorded in the database. A minimum follow-up of 60 months was required for the patients who are alive in the validation dataset so that their 5-year survival status is known. The primary endpoint is the overall survival (OS) time. Local recurrence (LR) time and distant metastases (DM) time are the secondary endpoints. The LR time was calculated from the time of surgery to the time when cancer recurrence was determined in the pelvis or anastomosis by physical examination, colonoscopy, or imaging studies. The DM time was defined from the time of surgery to the identification of distant recurrence. There were three times of massive follow-up for all off-records patients via mail or telephone in1996, 2002, and 2007.

### Statistical Model Creation

Kaplan-Meier plots and log-rank tests were performed for each potential predictive variable for the primary endpoint OS and the secondary endpoints LR and DM. Cox proportional hazards (PH) model was performed to develop the predictive model for OS. All decisions with respect to the grouping of the categorical variables and categorizing the continuous variables were made before modeling. These predictive models were the basis for the nomograms and the estimated probabilities of interest (e.g., 5-year OS) were calculated and presented in the nomograms.

### Model Validation

Each nomogram went through two validation procedures: internal validation using the study patients for the model creation and external validation using the independent validation patients. For each outcome variable, the predicted probability from the nomogram was compared with the actual status (e.g., alive or dead 5 years from surgery) for these uncensored observations. In addition, the Harrell’s concordance index (c-index) was calculated for each nomogram [17]. This index calculates the proportion of all usable patient pairs in which the predictions and the outcomes are concordant and has a similar interpretation to that of the AUC. All the above validation analyses were performed for the study patient data and the independent validation data.

All the statistical analyses were performed using R 3.0.1.

## Results

### Outcomes and survival analyses

Of the 833 patients with locally advanced rectal cancer in training group, 267 patients (32%) experienced local recurrence and/or distant metastases, and 263 patients (31.5%) died of cancer or other reasons up to our last follow-up. Of those alive, median follow-up time was 51 months. The 5-year LR, DM, OS probabilities (estimated using Kaplan-Meier method) for all patients were 22.3%, 32.7% and 63.8%, respectively.

Demographic and clinicopathologic variables that potentially predict OS, LR and DM were collected, including age, gender, tumor location, preoperative carcinoembryonic antigen level (CEA), tumor differentiation, tumor histopathology, number of metastatic lymph nodes, number of total sampled lymph nodes, lymphovascular invasion, perineural invasion, T classification, N classification and adjuvant treatment. For each outcome variable (LR, DM, and OS), univariate analysis identified statistically significant predictors in the demographic features, clinical features, pathological features and treatment modalities. 5-year local control, distant control and overall survival rates were provided for every category of each predictor with p-values obtained from the Log-rank tests (Table 1).

### Nomograms

**Figure 1:**
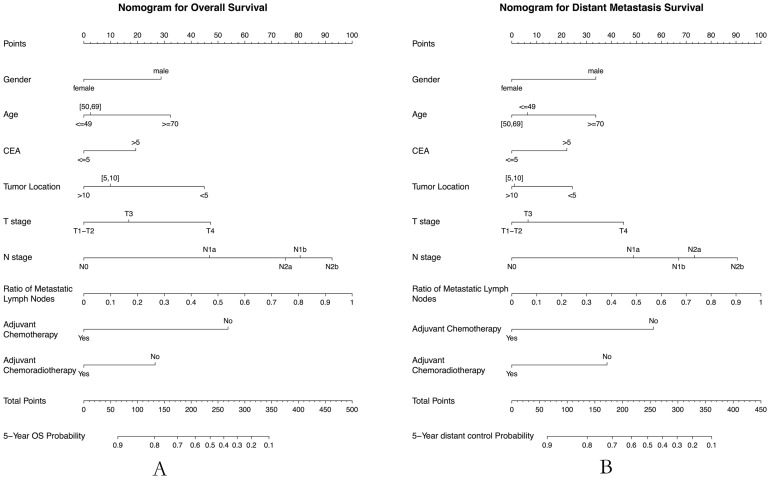
Nomograms developed for predicted 5-year overall survival (A) and distant control survival (B). Each variable value is assigned a score, and the sum of scores is converted to a probability of observed events in the lowest scale.

For the development of nomograms, all patients in the main dataset were included (N = 833), and the nomograms were validated using the external dataset (N = 84). Two nomograms for overall survival and distant metastases were successfully developed (Figure 1). The predictors included in the nomograms are gender, age (< = 49, 50–69, > = 70), tumor location (<5 cm, 5 cm-10 cm, >10 cm), adjuvant chemotherapy (No/Yes), adjuvant chemoradiotherapy (No/Yes), T classification (T1–T2, T3, T4), N classification (N0, N1a, N1b, N2a, N2b), CEA (< = 5, >5) and ratio of metastatic lymph nodes. Table 2 presents the hazard ratio (HR) with 95% CI and the pvalue for each predictor, and the c-index for the main dataset and the external dataset respectively. For OS prediction, the c-index was 0.76 in external validation, with a 95% CI of 0.67 to 0.86. Similarly, for DM prediction, the c-index was 0.73 (95% CI, 0.63–0.84). However, the nomogram for local recurrence prediction was not developed because of the poor c-index value in external validation (c-index, 0.6; 95% CI, 0.45–0.75).

**Table 2:** Multivariate analyses of 5-year outcomes: the final predictors for developing the nomograms.

Variable	Cox PH Regression			Nomogram	
	HR	95% CI	*p*-value	C-index	95% CI
**Distant Metastases**					
Gender					
Male vs Female	1.42	[1.07,1.88]	0.014		
Age (years)					
50–69 vs < = 49	0.94	[0.69,1.27]	0.672		
> = 70 vs < = 49	1.33	[0.92,1.93]	0.134		
Tumor location					
Mid ([5 cm, 10 cm]) vs Low (<5 cm)	0.79	[0.58,1.07]	0.122		
High (>10 cm) vs Low (<5 cm)	0.78	[0.46,1.31]	0.344		
Adjuvant chemotherapy					
Yes vs No	0.55	[0.41,0.74]	<0.0001		
Adjuvant chemoradiotherapy					
Yes vs No	0.67	[0.50,0,90]	0.008	Training Data: 0.68	[0.64,0.72]
pT classification				Validation Data:0.73	[0.63,0.83]
T3 vs T1–T2	1.07	[0.66,1.72]	0.781	Ten-fold Cross	
‘T4’ vs ‘T1–T2’	1.59	[1.03,2.47]	0.038	Validation	
pN classification				(Training Data): 0.65	
‘N1a’ vs ‘N0’	1.66	[1.05,2.62]	0.031		
‘N1b’ vs ‘N0’	2.00	[1.23,3.27]	0.005		
‘N2a’ vs ‘N0’	2.14	[1.21,3.80]	0.009		
‘N2b’ vs ‘N0’	2.56	[1.23,5.32]	0.012		
CEA					
>5 vs < = 5	1.26	[0.96,1.64]	0.093		
LNR					
Continuous*	1.11	[1.02,1.20]	0.013		
**Overall Survival**					
Gender					
Male vs Female	1.37	[1.06, 1.78]	0.017		
Age (years)					
50–69 vs < = 49	1.03	[0.77,1.36]	0.854		
> = 70 vs < = 49	1.42	[1.00,2.02]	0.049		
Tumor location					
Mid ([5 cm, 10 cm]) vs Low (<5cm)	0.68	[0.52, 0.90]	0.007		
High (>10 cm) vs Low (<5 cm)	0.61	[0.37, 1.01]	0.053		
Adjuvant chemotherapy					
Yes vs No	0.56	[0.42, 0.73]	<0.0001		
Adjuvant chemoradiotherapy					
Yes vs No	0.75	[0.57, 0.98]	0.033	Training data: 0.70	[0.66, 0.73]
pT stage				Validation data: 0.76	[0.67, 0.86]
‘T3’ vs ‘T1–T2’	1.20	[0.77, 1.87]	0.414	Ten-fold Cross	
‘T4’ vs ‘T1–T2’	1.68	[1.11,2.52]	0.013	Validation	
pN stage				(Training Data): 0.67	
‘N1a’ vs ‘N0’	1.67	[1.08, 2.59]	0.021		
‘N1b’ vs ‘N0’	2.42	[1.54, 3.79]	0.00012		
‘N2a’ vs ‘N0’	2.28	[1.33, 3.91]	0.0028		
‘N2b’ vs ‘N0’	2.75	[1.40, 5.44]	0.0035		
CEA					
>5 vs < = 5	1.24	[0.96, 1.59]	0.097		
LNR					
Continuous*	1.12	[1.03, 1.20]	0.0046		

Note: The concordance index (c-index) for the training and external validation are given for the nomogram as a performance measure; Tumor location was determined the distance from anal verge by preoperative colonoscopy or digital examination.

*LNR was analyzed as a continuous variable.

Abbreviations: HR, hazard ratio; PH, proportional hazards; c-index, concordance index; CEA, carcinoembryonic antigen; LNR, metastatic lymph nodes ratio.

### Predicted events within each AJCC stage classification

**Figure 2:**
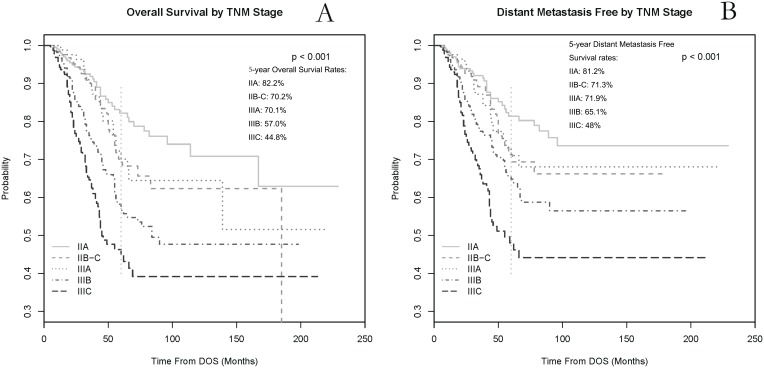
The overall survival (A) and distant metastases free (B) Kaplan-Meier probability curves within each stage (AJCC 7^th^ Edition) classification in locally advanced rectal cancer.

Within each AJCC stage (7^th^ Edition), the 5-year OS rates were 82.2% (stage IIA), 70.2% (stage IIB–C), 70.1% (stage IIIA), 57.0% (stage IIIB) and 44.8% (stage IIIC); and the 5-year DM rates were 19.8% (stage IIA), 28.7% (stage IIB–C), 28.1% (stage IIIA), 34.9% (stage IIIB), and 52.0% (stage IIIC), respectively. The Kaplan-Meier survival probability curves by AJCC stage were plotted for OS and DM in Figure 2. The overall log-rank tests for testing whether the survival curves are the same among all AJCC stage groups are significant for both OS and DM (p<0.001).

**Figure 3:**
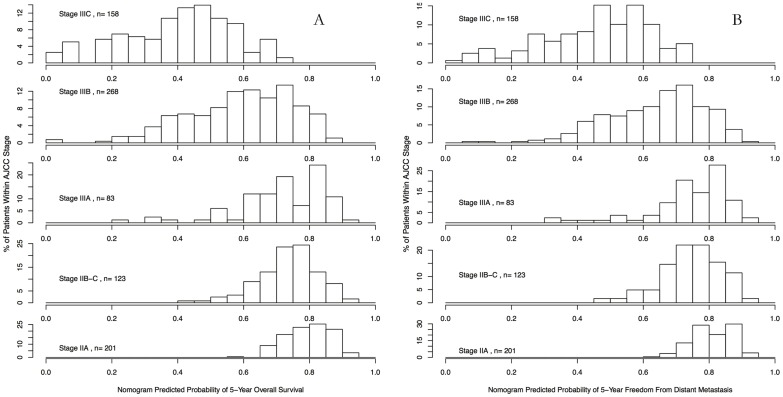
Histogram of nomogram-predicted 5-year overall survival rate (A) and distant control rate (B) within each subgroup of the 7^th^ edition of American Joint Committee on Cancer (AJCC) stage.

Based on our developed nomograms, the predicted probability of 5-year overall survival and distant control for each patient was computed, and the corresponding histograms were produced by AJCC stage classification from stage IIA to stage IIIC, respectively (Figure 3). The histograms showed that even within the same AJCC stage category, there are still a substantive amount of variability in terms of the predicted 5-year OS and DM-free probabilities, while in average the later stage patients have a smaller probabilities compared to earlier stage patients for both survival outcomes. Greater variations were observed for later stage patients (stage IIIB and IIIC) than earlier stage patients (stage IIA to IIIC) in terms of both 5-year OS and DM-free predicted probabilities.

## Discussion

In current study, for AJCC stage II–III (7^th^ edition) rectal cancers without neoadjuvant treatment, we have developed prognostic monograms with independent validation samples for predicting OS and DM, based on demographic, clinicopathological and adjuvant treatment information. Our models were developed using a 20-year period institutional database; during that time, neoadjuvant RT or CRT was not well applied in China. Our predictive models are helpful to support decision-making in clinical practice and follow-up protocols, especially in patients with rectal cancer who are preoperatively under-staged and undergo surgical resection first.

The purpose of treatment in rectal cancer is to potentially improve symptoms through local control, increase chance of cure, or prolong survival. Although the German Rectal Cancer Study Group established the significant improvements in local control and toxicity for patients with locally advanced rectal cancer treated with preoperative CRT [4], long-term follow-up and other clinical trials didn’t show benefit in overall survival and distant control for patients undergoing preoperative CRT[18]–[21]. A variety of factors ultimately influence a patient’s decision to receive preoperative CRT, such as proximal tumor location, suboptimal preoperative staging methods, inaccessible facilities for optimal radiotherapy, patient preference, and/or financial considerations. The potential benefits of receiving preoperative CRT must be carefully evaluated with the potential risks. Currently, there is no nationwide or international report about the accurate proportion of preoperative CRT in locally advanced rectal cancer. The US National Cancer Database (NCDB) reported that in 2008, 41% of patients with stage I–II rectal cancer received proctocolectomy with chemotherapy or radiotherapy, in which 80% of chemotherapy, which is mainly accompanied by radiotherapy, was delivered preoperatively. However, the percentage of preoperative CRT in stage II–III rectal cancer was not reported [2]. In Canada, only an average of 45% of stage II–III rectal cancers treated in 2007–2008 were reported to undergo preoperative RT or CRT in a Canadian nationwide cancer performance report [1], [22]. In Asian countries, much lower percentage of stage II–III rectal cancers undergo preoperative RT or CRT, as most surgeons in Asia do not usually recommend preoperative CRT for clinical T2 or T3 rectal cancers [3]. The wide variation in indications and clinical applications of neoadjuvant RT or CRT reflect the complexity of the disease, which should alert international rectal cancer expert organizations as well as health-care administrators. Therefore, in current clinical circumstance, there are still a great number of patients with locally advanced rectal cancer receiving curative surgical treatment prior to RT or CRT. Our study will help rectal cancer patients and physicians to pursue more individualized postoperative treatment according to their risks of disease control and survival expectations.

With the wide utilization of neoadjuvant CRT in clinical practice and randomized clinical trials, several studies focused on the outcome prediction in patients with combined modality treatment. Recently, a prediction nomogram was developed to predict local recurrence, distant metastases, and survival for patients with locally advanced rectal cancer treated with long-course chemoradiotherapy (CRT) followed by surgery in five European phase III clinical trials [16]. Postoperative ypT stage and ypN stage were most relevant to overall survival. However, as downstaged by preoperative CRT, the two most important prognostic factors (ypT and ypN classfications) could not be well applied to patients treated with curative surgery prior to adjuvant treatment. Otherwise, the decision of neoadjuvant CRT mainly relies on preoperative staging of the primary tumor. The accuracy of T and N stage by preoperative MRI or endorectal ultrasound varies, especially in N stage. A number of patients with locally advanced rectal cancer will be under-staged preoperatively and undergo surgery first. The postoperative treatment and outcome prediction for this group of patients are currently lacking. Moreover, although perioperative CRT or CT has been proved to be effective in rectal cancer, in real clinical circumstance, there are still a part of patients with locally advanced rectal cancer undergoing surgery alone. According to a large-scale population-based study through the California Cancer Registry, there were still 33% and 18.6% of patients with stage II and stage III rectal cancer undergoing surgery alone from the year 1994 to 2008 [23]. Similarly, 57.4% and 13.0% of patients with stage II and stage III rectal cancer underwent surgery alone in our study. Currently, we are lacking of studies in defining characteristics of patients who have good outcomes without neoadjuvant therapy, particularly with surgery alone. Our nomogram provides a helpful tool for identifying patients with good outcomes if they were preoperatively under-staged and underwent surgery first. Meanwhile, as preoperative CRT contributed small improvements in overall survival and distant metastases, our study provided helpful tools and comparable dataset for predicting patients’ distant control and overall survival in locally advanced rectal cancer with multiple treatment modalities.

The goal of our study is to develop monograms to predict overall survival and distant metastases for patients without preoperative treatment. To our knowledge, using the 7^th^ edition of AJCC staging system was the first predicting model for OS and distant control in rectal cancer (Figure 2), especially in Asian patients who were less represented in the AJCC stage system. Similar survival differences among different AJCC stage categories were observed in our patient cohort, as compared with Surveillance, Epidemiology, and End Results (SEER) population-based data [24]. Postoperative T stage and N stage were still most significant factors to predict OS and DM rates. However, from the predicted outcomes based on our nomograms, heterogeneities in the risk of death and distant metastases still largely existed within each sub-category stage from stage IIA to stage IIIC. Specifically, from the histograms in Figure 3, the variability of predicted OS and DM rates was observed greater in patients in stage IIIB and IIIC than patients in stage IIA–IIIA. This suggests that the prediction value of OS and DM may be better in patients with stage IIIB and IIIC rectal cancer when adding these demographic and clinicopathological variables which were not included in TNM staging system; while for patients with stage IIA to IIIA, molecular markers (eg. microsatellite instability, loss of heterozygosity, etc.), rather than adding more clinicopathological variables, may be benefit to further improve the accuracy of outcome prediction. By integrating important demographic and clinicopathological features, our nomogram helped further individualize the outcome prediction based on current TNM staging system. More personalized postoperative treatment may be utilized for preoperatively under-staged patients with rectal cancer in the same AJCC stage.

In addition to the TN stage, metastatic lymph nodes ratio (LNR) was reported to be a reliable prognostic factor both in colon and rectal cancer[25]–[28]. However, utilization LNR in clinical practice is relatively difficult, as optimal cut-off of the continuous LNR value has not been established. We also found LNR was one of most important prognostic factors for predicting DM and OS, in addition to patients' N stage. LNR was treated as a continuous variable in our predicting nomograms, which contributed to improve the performance of our model in predicting patients’ survival outcomes. Data from the five European trials found small but statistical significant improvement in distant control for patients with neoadjuvant CRT [16]. A recent meta-analysis of 21 randomized controlled trials from 1975 to 2011 concluded that adjuvant 5-Fu-based chemotherapy was beneficial for rectal cancer patients in improving overall survival and disease-free survival [29]. However, the benefit of adjuvant chemotherapy after combined treatment of rectal cancer is still not well defined in single randomized trials [4], [19], [21]. In our patient cohort, we only found improvements in local control in patients with any adjuvant treatment, compared with no adjuvant treatment. Further clinical trials are needed to explore the effect of adjuvant chemotherapy (single agent or combination) in improving distant control and overall survival.

Currently, there are emerged debates about adding adjuvant radiotherapy to node positive patients who receive surgical treatment first because of under-staged disease by preoperative imaging. Although randomized clinical trials proved the improvement of local control in node positive rectal cancer [30], [31], the risks of treatment toxicities and decremented quality of life limited its clinical use [32], [33]. A predicted nomogram for local recurrence including demographic and clincopathological variables may help physicians to choose patients who may benefit more from adjuvant radiotherapy. In our study, improved local control was observed in patients with any adjuvant treatment in univariate analysis, and the most optimal local control were observed in patients with adjuvant chemoradiotherapy followed by chemotherapy (Table 2). Unfortunately, our study was not able to develop a reliable nomogram for predicting local recurrence. Treatment variations in adjuvant setting, heterogeneous data, lacking of statistical power, less events in the validation group may be attributed to this. Further studies are needed to develop a reliable predictive model for local recurrence in preoperatively under-staged patients.

As a retrospective study, there are other limitations: detailed regimens of adjuvant chemotherapy could not be clearly provided for each patient; techniques of radiotherapy are changing over the 20 years; detailed information of recurrence may be unclear for part of patients, as well as loss of follow-up problems. However, our study still provides a valuable tool to help clinicians manage under-staged patients with rectal cancer who undergo surgery first. Further study is needed to provide optimal postoperative treatment for these patients.

## Conclusions

The prognostic nomograms integrated demographic and clinicopathological factors to account for tumor and patient heterogeneity, and thereby provided a more individualized outcome prognostication than that by the AJCC staging system alone. Our individualized prediction nomograms could help physicians counsel and advise patients about their personalized treatment strategies and follow-up protocols, especially in patients with preoperatively under-staged rectal cancer.
